# The Effectiveness of Improving Infectious Disease–Specific Health Literacy Among Residents: WeChat-Based Health Education Intervention Program

**DOI:** 10.2196/46841

**Published:** 2023-08-09

**Authors:** Yusui Zhao, Shuiyang Xu, Xuehai Zhang, Lei Wang, Yu Huang, Shuxian Wu, Qingqing Wu

**Affiliations:** 1 Zhejiang Provincial Center for Disease Control and Prevention Department of Health Education Hanghzhou China

**Keywords:** effectiveness, health education, infectious disease-specific health literacy, intervention, WeChat

## Abstract

**Background:**

Infectious disease–specific health literacy (IDSHL) has become an important determinant of infectious disease incidence. It can not only reduce the incidence of re-emerging infectious diseases, but also effectively prevent the emergence of new infectious diseases such as COVID-19. WeChat, as a new media, has been proven to greatly reduce the chance of infectious diseases spreading from person to person, especially in case of respiratory infections. However, there is currently no concrete health education invention program to improve IDSHL using a WeChat public account.

**Objective:**

The aims of this study were as follows: (1) to determine the IDSHL of the population in Zhejiang, China; (2) to develop a health education program for the improvement of IDSHL using a WeChat public account; and (3) to evaluate the effectiveness of the health education program that was implemented in the prevention of infectious disease outbreaks.

**Methods:**

We used a standardized questionnaire, which consisted of 28 closed-ended questions, to measure the level and score of IDSHL before and after intervention. A multiple-stage stratified random sampling technique was used to select study participants from Zhejiang province in China, who were further divided randomly into 2 groups: the intervention and control groups. From July 2014 to January 2015, a WeChat-based health education intervention program was carried out on the intervention group. Standard descriptive statistics and chi-square and *t* tests were conducted to analyze the data.

**Results:**

A total of 3001 residents participated in the baseline survey of this study. At baseline, participant IDSHL rates were 73.29% and 72.12% for the intervention and control groups, respectively (Χ^2^_1_=0.5; *P*=.50). After 7 months of intervention, 9.90% (297/3001) of participants dropped out of the study. Of the lost participants, 119 were from the intervention group and 178 were from the control group. There were significant differences between follow-up and lost participants with respect to age (*P*=.04), marital status (*P*=.02) and occupations (*P*=.002). After intervention, the intervention group scores in the different domains were higher than those in the control group (infectious disease–related knowledge, prevention, management, or treatment, identification of pathogens and infection sources, and cognitive ability). There were significant improvements in the IDSHL of participants in both the intervention and control groups (Χ^2^_1_=135.9; *P*<.001 vs Χ^2^_1_=9.1; *P*=.003), and there was a greater change in the IDSHL among the intervention group participants than among the control group participants (1230/1359, 90.51% vs 1038/1359, 77.17%).

**Conclusions:**

The health education intervention program using a WeChat public account proved to be an effective, feasible, and well-accepted means to improve the IDSHL of the general population. In the future, this health education intervention program can be used as a reference for prevention and treatment of infectious diseases.

## Introduction

Health literacy (HL) is defined as “the degree to which individuals have the capacity to obtain, process, and understand basic health information and services needed to make appropriate health decisions” [1]. Infectious disease–specific health literacy (IDSHL) focuses on 3 core principles, cognition, decision-making, and self-efficacy, to prevent or treat infectious disease [2]. Compared to their counterparts with high HL, individuals with low HL for one or several specific diseases have been shown to have worse health outcomes [3] and have been associated with less knowledge and skills regarding communicable diseases [4], reduced adoption of protective behaviors such as hand hygiene practices [5], and poorer health and quality of life [6]. Accordingly, IDSHL has been recognized as a very important determinant of infectious disease incidence and outcomes.

In recent years, most studies regarding HL have focused on patients in high-income countries with specific diseases such as HIV and AIDS [7], diabetes [8], kidney disease [9], and heart failure [10], rather than HL in the general population. Compared to developed countries, China continues to have a high incidence of infectious diseases, such as HIV and AIDS, sexually transmitted diseases, viral hepatitis, and tuberculosis [11]. Although infectious disease mortality has decreased substantially (>90%) in recent years [12], China is seeing the re-emergence of previously prevalent infectious diseases (HIV and AIDS, sexually transmitted diseases, viral hepatitis, tuberculosis, and rabies) as well as the emergence of new infectious diseases, such as severe acute respiratory syndrome (SARS), the novel avian influenza A (H7N9) virus, and COVID-19 [13-15].

Zhejiang is a province in southeastern China that had a population of 52 million people at the end of 2009. It has seen the second-largest influx of migrant workers in China and is currently a large province of migrant workers. These workers have left their homes to live in overcrowded accommodations with poor sanitation, and they are not entitled to certain rights, including open employment opportunities, free education, social welfare, and medical benefits. Therefore, this greatly increases the risk of an outbreak of infectious disease.

Compared to traditional health education, the emergence of new media provides greater convenience for the sharing of information about health and medical conditions as well as health messages [16]. The use of new media to carry out health education not only makes health information easier to obtain and share but also avoids direct contact between people. This greatly reduces the chance of infectious diseases spreading from person to person, especially in the case of respiratory infections [17]. Following Facebook and Twitter’s success in medical education in Western universities [18], WeChat (Chinese pinyin: Wei Xin) is a new media app that is currently recognized as the best social networking site to use for a similar purpose, given that its users span all ages and professions in China [19]. As a free instant messaging app for smartphones, WeChat enables the exchange of text, voice, picture, and video information between individuals and groups through mobile phones. There are currently an estimated 963 million WeChat users in China [20]. As a result, WeChat has been widely used in the medical field and has had a positive influence [21]. To date, most studies have focused on using WeChat public accounts for applications in clinical medicine or other areas [22]. However, few studies have focused on public health education using a WeChat public account to improve IDSHL.

Thus, the aims of this study were (1) to identify the IDSHL level of residents of Zhejiang Province, (2) to develop a health education program for the improvement of IDSHL using a WeChat public account, and (3) to evaluate the effectiveness of the health education program that was implemented to prevent infectious disease outbreaks.

## Methods

### Participants and Survey Design

Between November and December 2014, a cross-sectional survey was conducted in Zhejiang, China. A multiple stage–stratified random sampling technique was used to select study participants. First, counties in Zhejiang were categorized into 3 strata by socioeconomic development (good, fair, and poor). The top 25% of the county’s gross domestic product (GDP) is classified as “good,” while 25%-75% are “fair,” and others are considered “poor.” If those with good GDP are in the first strata, those with fair GDP are in the second strata, and those with poor GDP are in the third strata. A total of 2 counties were randomly selected from each group. Second, 2 towns or streets were randomly selected from all of the candidate towns or streets in each selected county, and 1 residential committee or village (about 500-1500 households) was sampled randomly from each town or street. Third, from each residential committee and village, a systematic random sampling technique was used to select 250 households from the household registration list. The family member with the closest birth date to the survey date was surveyed. Finally, we recruited 3023 residents from 6 surveillance sites in Zhejiang Province. Eligibility criteria included (1) age 15 to 69 years, (2) ability to read or communicate, and (3) accessibility of participants to researchers. A total of 3001 residents were recruited for our baseline survey. After 7 months of intervention, a second survey was conducted. For the control group, inclusion criteria included (1) regular residence in their residential committees or villages for the 7-month period and (2) full and voluntary participation in the regular health education intervention activities. For the intervention group, in addition to the inclusion criteria of the control group, the inclusion criteria also included (3) following our WeChat public account and not canceling it during the period and (4) being able to check the messages and lectures on infectious disease prevention and treatment pushed by the WeChat public account on time.

The survey participants’ IDSHL was assessed using the IDSHL instrument developed by Tian et al [2] in 2014 and applied to measure the level of Chinese residents’ IDSHL. From July 2014 to January 2015, after the initial IDSHL assessment, a WeChat-based health education intervention program was implemented. A total of 2 counties in each stratum were divided randomly into 2 groups: the intervention group and the control group. There were 1480 participants in the intervention group and 1523 in the control group.

**Figure 1:**
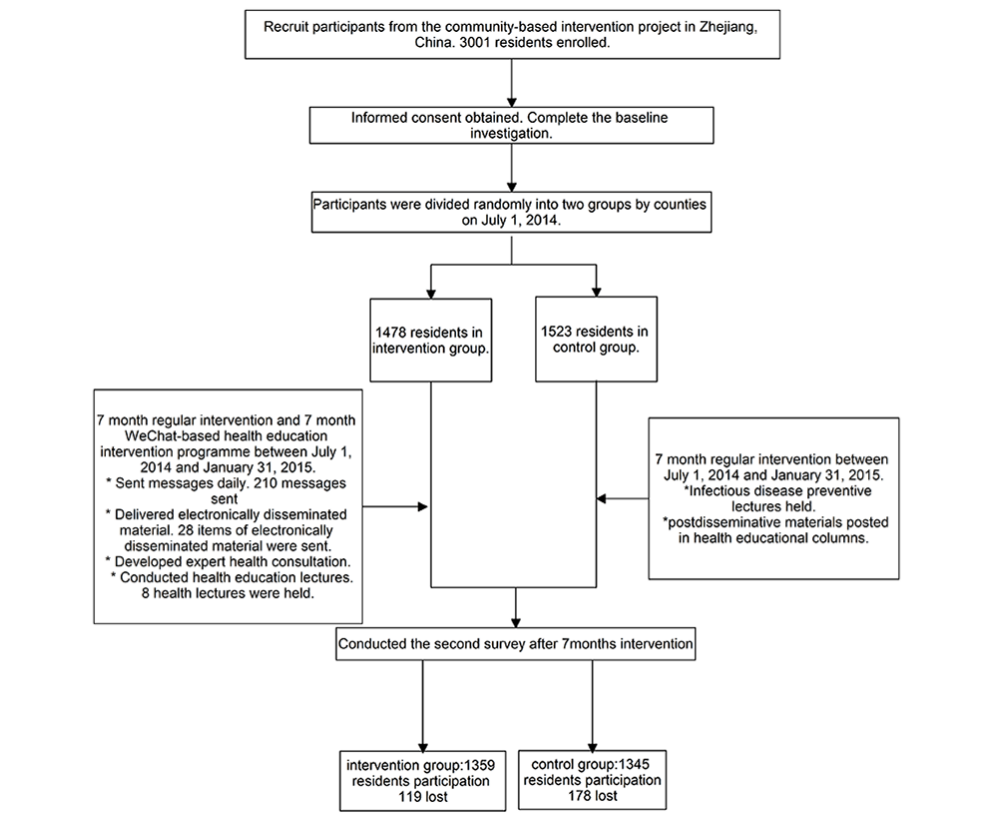
Study protocol flowchart.

A postintervention IDSHL assessment was conducted in order to note any changes in IDSHL among the study population. The study’s protocol is reflected in the flowchart (Figure 1).

### IDSHL Assessment

The instrument, which included 28 items, was developed by Tian et al [2] in 2014 and was divided into subscale 1 (containing 22 items) and subscale 2 (containing 6 items). The IDSHL was obtained through 22 items in subscale 1 which included 4 domains: infectious disease–related knowledge and values, including transmission of infectious diseases, infectious disease prevention, management or treatment of infectious diseases, and identification of pathogens and infection sources. Subscale 2 mainly involved cognitive ability, including 3 aspects: ability to access health information, understanding of infectious disease–related information, and capacity to use health information. We took the reciprocal of the correct answer rate of each item as the difficulty factor of the item. At the same time, each difficulty coefficient is used as the score of each item. In the end, the total score of the instrument was 38.62. Each of the 22 items had a content validity index >0.8 and the overall Cronbach α coefficient was .84. Considering receiver operating characteristic (ROC) curve sensitivity (77.3%) and specificity (45.1%), the cut-off value of adequate IDSHL was scores of ≥16.74 with 38.62 as the total score.

### Intervention

#### Regular Intervention

After the community-based intervention was developed, it was implemented between July 1, 2014, and January 31, 2015. Both the control and intervention groups received regular health education intervention. The regular health education intervention activities are those incorporated into the national basic public health service program, which is a government-mandated program that each community health service center is required to carry out on the community residents in its jurisdiction. It included infectious disease preventive lectures every 3 months and the dissemination of materials posted in health educational columns and updated every 3 months. The regular health education intervention was carried out by a responsible doctor in the community.

#### WeChat-Based Health Education Intervention Program

The intervention contents included infectious disease preventive measures, basic knowledge and skills to become proficient in understanding infectious disease–related information, as well as basic communication skills for acquiring, accessing, and practicing newly acquired health information. The electronically disseminated materials, lecture materials, and health messages were designed by experts from the Chinese Health Education Center to attract interest from participants. The WeChat-based intervention program was carried out by trained health service educators from the Community Health Service Center. Electronic materials that were disseminated, such as posters and booklets, were designed by health education experts and included critical information about infectious diseases and their prevention and treatment. The health service educators were trained by health education experts in topics such as infectious disease prevention, health education, and health communication. Each participant in the intervention group received the program for 7 months. The materials and courses involved were only applied to the intervention group during the intervention process. The WeChat-based health education intervention program included the following:

1. Messages related to infectious disease treatment and prevention were sent daily by intervention managers on a WeChat public account, and everyone in the intervention group could receive them. A total of 210 messages were sent.
2. Electronically disseminated materials, such as posters and booklets, were sent weekly by intervention managers on a WeChat public account, and everyone in the intervention group could receive them. A total of 28 electronically disseminated materials were sent.
3. Participants in the intervention group could ask questions relating to infectious disease prevention and treatment that were only visible to the trained health educators, and the participants received responses within 24 hours.
4. Lectures on infectious disease prevention and treatment were presented to the intervention group every month through a WeChat public account. A total of 8 health lectures were held.

### Data Analysis

Statistical analyses were performed using SPSS (version 18.0; IBM Corp). A total of 3 sets of analyses were performed. First, chi-square and *t* tests were used to test statistical differences in the demographic characteristics and IDSHL between the follow-up group and the lost group in both the intervention and control groups. Second, chi-square analyses were used to test differences in IDSHL between the 2 groups. Third, descriptive statistics were used to analyze participation for the intervention project. A *P* value less than .05 was considered statistically significant.

### Ethics Approval

This study followed the tenets of the Declaration of Helsinki. Written consent was obtained from all participants or from their caregivers or guardians on their behalf. The study was also approved by the ethics committee of the Zhejiang Provincial Center for Disease Control and Prevention (20140100).

## Results

### Sociodemographic Characteristics of the Study

A total of 3001 residents participated in our baseline survey. Of these, 1545 (51.48%) were women. The ages of the respondents ranged from 18 to 69 years, with an average age of 43.62 (SD 9.91) years. Most participants were Han Chinese (2980/3001, 99.60%) and married (2785/3001, 92.80%). About half of the participants had completed middle school (1362/3001, 45.38%). Additionally, approximately half were migrant workers (1540/3001, 51.32%). There were significant differences between the intervention group and control group with regard to occupation (*P*<.001). Compared with the intervention group, the proportion of migrant workers and agency or institutional personnel was higher in the control group (Table 1).

**Table 1:** Sociodemographic characteristics of participants in different groups between intervention and control groups.

Content and group		Baseline total (N=3001), n (%)	Intervention group (N=1478), n (%)	Control group (N=1523), n (%)	Chi-square (*df*)	*P* value
**Gender**					0.3 (1)	.57
	Male	1456 (48.52)	766 (51.83)	805 (52.86)		
	Female	1545 (51.48)	712 (48.17)	718 (47.14)		
**Age (years)**					4.7 (3)	.20
	18-29	298 (9.93)	146 (9.88)	152 (9.98)		
	30-39	694 (23.13)	319 (21.58)	375 (24.62)		
	40-49	1078 (35.92)	535 (36.20)	543 (35.65)		
	50-69	931 (31.02)	478 (32.34)	453 (29.74)		
**Ethnicity**					0.003	.96
	Han	2989 (99.60)	1472 (99.59)	1517 (99.61)		
	Minority	12 (0.40)	6 (0.41)	6 (0.39)		
**Education**					8.4 (4)	.08
	Illiterate	231 (7.70)	113 (7.65)	118 (7.75)		
	Elementary school	779 (25.96)	383 (25.91)	396 (26)		
	Middle school	1362 (45.38)	642 (43.44)	720 (47.28)		
	High school	461 (15.36)	247 (16.71)	214 (14.05)		
	College or university	168 (5.60)	93 (6.29)	75 (4.92)		
**Marital status**					1.8 (2)	.41
	Unmarried	123 (4.10)	62 (4.19)	61 (4.01)		
	Married	2785 (92.80)	1364 (92.29)	1421 (93.3)		
	Divorced	93 (3.10)	52 (3.52)	41 (2.69)		
**Occupation**					57.6 (6)	<.001
	Migrant workers^a^	1540 (51.32)	736 (49.8)	804 (52.79)		
	Agency or institutional personnel^b^	246 (8.20)	94 (6.36)	152 (9.98)		
	Businesspeople^c^	807 (26.89)	416 (28.15)	391 (25.67)		
	Students	24 (0.80)	14 (0.95)	10 (0.66)		
	Unemployed	46 (1.53)	43 (2.91)	3 (0.2)		
	Retirees	12 (0.40)	10 (0.68)	2 (0.13)		
	Other^d^	326 (10.86)	165 (11.16)	161 (10.57)		
**2-week morbidity^e^**					0.015 (1)	.90
	Yes	424 (14.13)	210 (14.21)	214 (14.05)		
	No	2577 (85.87)	1268 (85.79)	1309 (85.95)		

^a^Migrant workers refers to laborers still census-registered in rural areas, entering county laborers, and working in nonagricultural industries for 6 months or more locally or off-site.

^b^Agency or institutional personnel refers to all state organs, state-owned enterprises, institutions, and other public officials engaged in official duties according to law.

^c^Businesspeople refers to personnel engaged in commercial, catering, tourism, entertainment, transportation, medical assistance, social, and residential services.

^d^The “other” category includes unemployed people and those with occupations other than those already listed.

^e^2-week morbidity refers to the incidence of infectious diseases affecting the respondents in the past 2 weeks.

### Comparison of Sociodemographic Characteristics Between Follow-Up and Lost Participants

After 7 months of intervention, we evaluated the eligibility of those who completed the baseline survey for inclusion in the postintervention survey. According to this evaluation, 297 residents were no longer part of the study, resulting in a response rate of 90.10% (2704/3001). Of the lost participants, 119 were from the intervention group and 178 were from the control group. There were marginally significant differences between follow-up and lost participants with respect to age (*P*=.04). There were more married people in the follow-up compared to the lost group (*P*=.02). Compared with the lost group, the proportion of migrant workers was higher in the follow-up group (*P*=.002; Table 2).

**Table 2:** Comparison of sociodemographic characteristics between the follow-up group and those lost to follow-up.

Content and group		Follow-up (N=2704), n (%)	Lost to follow-up (N=297), n (%)	Chi-square (*df*)	*P* value
**Gender**				0.004 (1)	.95
	Male	1415 (52.33)	156 (52.53)		
	Female	1289 (47.67)	141 (47.47)		
**Age (years)**				8.3 (3)	.04
	18-29	259 (9.58)	39 (13.13)		
	30-39	637 (23.56)	57 (19.19)		
	40-49	981 (36.28)	97 (32.66)		
	50-69	827 (30.58)	104 (35.02)		
**Ethnicity**				0.03 (1)	.86
	Han	2693 (99.59)	296 (99.66)		
	Minority	11 (0.41)	1 (0.34)		
**Education**				4.5 (4)	.34
	Illiterate	206 (7.62)	25 (8.42)		
	Elementary school	702 (25.96)	77 (25.93)		
	Middle school	1216 (44.97)	146 (49.16)		
	High school	424 (15.68)	37 (12.46)		
	College or university	156 (5.77)	12 (4.04)		
**Marital status**				7.7 (2)	.02
	Unmarried	105 (3.88)	18 (6.06)		
	Married	2521 (93.23)	264 (88.89)		
	Divorced	78 (2.88)	15 (5.05)		
**Occupation**				20.8 (6)	.002
	Migrant worker	1357 (50.18)	183 (61.62)		
	Agency or institutional personnel	219 (8.1)	27 (9.09)		
	Businesspeople	744 (27.51)	63 (21.21)		
	Students	21 (0.78)	3 (1.01)		
	Unemployed	45 (1.66)	1 (0.34)		
	Retirees	12 (0.44)	0 (0)		
	Others	306 (11.32)	20 (6.73)		
**2-week morbidity**				0.3 (1)	.59
	Yes	379 (14.02)	45 (15.15)		
	No	2325 (85.98)	252 (84.85)		

### Effectiveness of WeChat-Based Health Education Intervention Program

Before intervention, there were no significant differences between the intervention group and control group with respect to subscale 1 scores, its 4 domains, and subscale 2 scores, except for the infectious disease prevention domain score (2-tailed t_2702_=2.451; *P*=.01). After intervention, the intervention group scores in the different domains were higher than those in the control group—infectious disease–related knowledge of the transmission of infectious diseases (8.31, SD 2.94 vs 7.17, SD 3; 2-tailed t_2702_=9.923; *P*<.001); infectious disease prevention (8.62, SD 1.92 vs 7.72, SD 2.26; 2-tailed t_2702_=11.033; *P*<.001); management or treatment of infectious diseases (5.47, SD 2.65 vs 4, SD 2.67; 2-tailed t_2702_=14.408; *P*<.001); identification of pathogens and infection sources (4.43, SD 2.55 vs 3.13, SD 2.36; 2-tailed t_2702_=13.838; *P*<.001); subscale 1 (26.83, SD 7.25 vs 22.02, SD 7.21; 2-tailed t_2702_=17.276; *P*<.001); and cognitive ability. According to Tian et al [2], cognitive ability included 3 aspects: ability to access health information, understanding of infectious disease-related information, and capacity to use health information (5.62, SD 2.92 vs 4.40, SD 2.61; 2-tailed t_2702_=11.392; *P*<.001; Table 3).

**Table 3:** Comparison of different subscale scores between the 2 groups, before and after intervention.

Subscale	Domain	Before intervention scores		2-tailed *t* test (*df*)	*P* value	After intervention scores		2-tailed *t* test (*df*)	*P* value
		Intervention group, mean (SD)	Control group, mean (SD)			Intervention group, mean (SD)	Control group, mean (SD)		
**Subscale 1**									
	D^1a^	7.47 (3.18)	7.43 (3.27)	0.37 (2702)	.71	8.31 (2.94)	7.17 (3.00)	9.92 (2702)	<.001
	D^2b^	7.67 (2.33)	7.45 (2.47)	2.45 (2702)	.01	8.62 (1.92)	7.72 (2.26)	11.03 (2702)	<.001
	D^3c^	3.81 (2.70)	3.98 (2.65)	1.66 (2702)	.20	5.47 (2.65)	4.00 (2.67)	14.41 (2702)	<.001
	D^4d^	2.76 (2.36)	2.85(2.25)	1.01 (2702)	.31	4.43 (2.55)	3.13 (2.36)	13.84 (2702)	<.001
Total		21.72 (7.81)	21.7 (7.91	0.04 (2702)	.97	26.83 (7.25)	22.02 (7.21)	17.28 (2702)	<.001
Subscale 2	D^5e^	4.23 (2.85)	4.27 (3.20)	0.37 (2702)	.71	5.62 (2.92)	4.40 (2.61)	11.39 (2702)	<.001

^a^D^1^: Infectious disease-related knowledge and values including transmission of infectious diseases.

^b^D^2^: Infectious disease prevention.

^c^D^3^: Management or treatment of infectious diseases.

^d^D^4^: Identification of pathogens and infection sources.

^e^D^5^: Cognitive ability.

**Figure 2:**
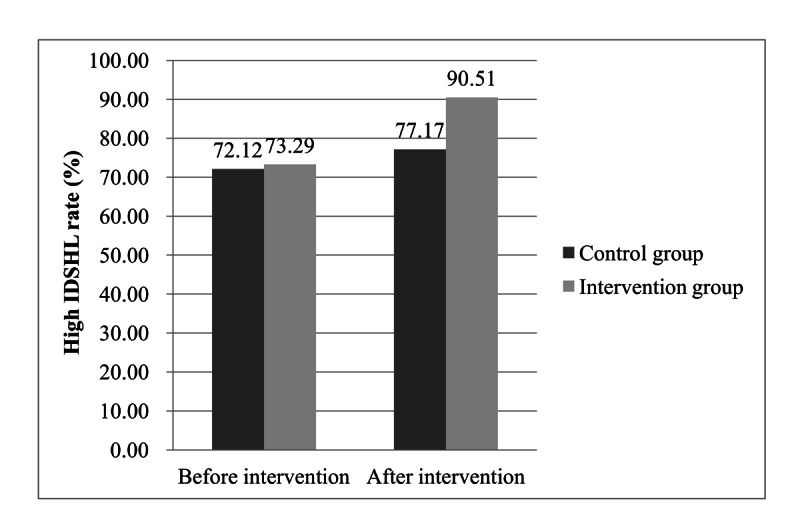
Comparison of changes in IDSHL between the intervention and control groups.

Before intervention, there was no significant difference between the intervention group and control group with respect to IDSHL (73.29% vs 72.12%; Χ^2^_1_=0.5; *P*=.50). After intervention, there were significant improvements in IDSHL for participants in both the intervention and control groups (Χ^2^_1_=135.9; *P*<.001 vs Χ^2^=9.1; *P*=.003). There was a greater change in IDSHL among the intervention group participants than among control group participants (90.51% vs 77.17%). Figure 2 shows the changes in the high IDSHL rate between the intervention and control groups.

## Discussion

### Overview

Since the start of the 21st century, China has faced 2 infectious disease challenges: the re-emergence of previously prevalent infectious diseases [23,24] and the emergence of new infectious diseases [13]. After the outbreak of SARS, the Chinese government strengthened the country’s infectious disease surveillance to detect infectious diseases in a timely fashion [25]. Compared to 1990, the incidence of infectious diseases, as well as the mortality and morbidity arising from them, has declined despite a growing population [26]. Inadequate HL has been associated with a reduction in the adoption of infection prevention–related behaviors, such as immunization [3]. HL is a very important infectious disease preventive measure [27]. Accordingly, efforts to improve IDSHL are necessary.

The measure used in our study was developed by Tian et al [2] whose project was funded by the National Sci-Tech Plan Project (code number 2013BAI06B06). Before Tian et al’s work, there were 2 similar instruments: one was a skills-based instrument [28] for measuring the HL of respiratory infectious diseases, and the other was an assessment of adults’ knowledge and skills regarding communicable diseases [4]. Taking into account the scientific and comprehensive nature of Tian et al’s [2] measure, it was applied to measure the level of IDSHL in Tibet [29] and Hubei Province, China. We selected this measure for the study in our province, and we found that the IDSHL rate of Zhejiang residents was higher when compared to another study of Beijing residents (49%) [4]. The main cause of this may be the use of different methods of measuring IDSHL. In this study, we not only focused on the knowledge and adoption of preventive measures and behaviors but also included aspects relating to management and treatment of infectious disease and participant cognitive ability in terms of content.

Traditional health education lectures have played a significant role in changing people’s health knowledge, awareness, and attitudes [30]. WeChat public accounts provide a new means of propagating information using IT technology and frequent multimedia messages for better communication among users. In this study, we evaluated the eligibility of those who completed the baseline survey for inclusion in the postintervention survey. The results showed that 297 residents were lost to follow-up, with a loss rate of 9.90%, which was less than 10%, and we judged the overall selection bias to be small. Next, we compared the differences in sociological characteristics between the follow-up group and those lost to follow-up. The results showed that there were no statistical differences between the 2 groups except for age (*P*=.04), marital status (*P*=.02), and occupation (*P*=.002). Ultimately, we think implementing a health education intervention program for prevention and treatment of infectious diseases in residents through WeChat proved effective in improving IDSHL in Zhejiang. WeChat can therefore be considered an important health promotion tool, especially during infectious disease epidemics such as COVID-19. Since March 11, 2020, when the World Health Organization declared the outbreak of COVID-19 a pandemic, the virus has infected more than 93 million people and killed over 2 million in more than 188 countries [31]. Available evidence implicates airborne transmission of SARS-CoV-2, the virus that causes COVID-19, through aerosols as a potential route for the spread of the disease [32]. The most effective way to prevent COVID-19 is to avoid human contact as much as possible. New media such as WeChat could not only improve residents’ HL levels of infectious diseases but also avoid person-to-person contact and the possibility of infection [33]. Despite our government’s increasing emphasis on health education, more health education professionals and research funds have been devoted to related research in the health education field. However, there are still many obstacles to implementing our intervention on a larger scale. For example, WeChat public accounts lack popularity, and health education professionals lack capacity.

### Limitations

This study had a few limitations. First, the representativeness of our study population compared to the general Chinese population may be affected by sampling. In this study, the participants were recruited from 6 counties in 1 province. Second, the study, which recruited participants through a WeChat public account, had a bias in favor of young Chinese people. Third, whether the effect of the intervention can be sustained or whether there is a causal relationship between IDSHL, and infectious disease incidence needs to be considered in further studies. Finally, we tried our best to minimize the loss to follow-up rate of the investigated subjects after intervention in terms of quality control. Although we kept the loss to follow-up rate within 10%, it was still possible to affect the results of the study.

### Conclusions

We found higher IDSHL levels in participants from Zhejiang Province compared to previous studies. After the intervention, the IDSHL scores of both the intervention and control groups improved significantly, though the improvement was greater in the intervention group. The health education intervention program using a WeChat public account proved to be effective, feasible, and well-accepted as a means to improve IDSHL of the general population. In the future, this health education intervention program can be used as a reference for the prevention and treatment of infectious diseases. Despite the limitations of this study, it still makes a significant contribution to the literature and has implications for society or the field at large.

### Data Availability

Anonymized data from this paper are available for research purposes on request to the corresponding author. Broader public data sharing for this study has been restricted by the ethics committee of the Zhejiang Provincial Center for Disease Control and Prevention because the consent obtained from the participants does not cover the unlimited public sharing of the data.

## Appendix

Multimedia Appendix 1 Questionnaire on Infectious Disease-Specific Health Literacy.

## Abbreviations

GDP: Gross Domestic Product
H7N9: novel avian influenza A
HL: health literacy
IDSHL: infectious disease–specific health literacy
ROC: receiver operating characteristic
SARS: severe acute respiratory syndrome
