# Supplementary material for: The Effectiveness of Improving Infectious Disease–Specific Health Literacy Among Residents: WeChat-Based Health Education Intervention Program
Source: JMIR Form Res. 2023 Aug 9;7:e46841. doi: 10.2196/46841 (PMC10448287; doi:10.2196/46841)
Supplement: Multimedia Appendix 1 [file formative_v7i1e46841_app1.docx]

Questionnaire on Infectious Disease-Specific Health Literacy

coding：□□□□□□□

**Instructions for filling out the questionnaire**

Please fill in the box after each question with the serial number of the option you selected, such as:

2. Gender： ①male  ②female 
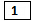


**Introduction and informed consent**

Hello, we are the staff of Zhejiang Provincial Center for Disease Control and Prevention. In order to understand your infectious disease-specific health literacy, we need to ask you a few questions, please provide some personal information, contact information, answer some questions about the prevention and treatment of infectious diseases. This information will be only to facilitate the future to find you and give you scientific health guidance. The content involved in the investigation will be kept confidential for you and will not be disclosed to any third party. So please answer truthfully. Participation in this survey will be completely voluntary, and you may refuse to answer any questions you are not willing to answer, and you can terminate the survey at any time if you wish to do so in the survey. By participating in this survey, you can acquire knowledge and information on the prevention of infectious diseases, and help relevant departments formulate strategies and measures for the prevention and control of infectious diseases. So as to effectively prevent infectious diseases and better protect everyone. The survey will take you about 15 minutes. I will give you a small souvenir after the survey. If you agree to participate in the survey, please sign and cooperate with us to complete the survey.

Thank you for your support!

**Respondent's signature after informed consent:**

**Sociodemographic characteristics**

1.**Name**：

1. **Gender**：  ①male     ②female  **□**
2. **Age**：_________

4.**Contact information**

Mobile： ； WeChat No.：

5.**Ethnicity**：  ①Han ②Minority **□**

6.**Education**：  **□**

①Illiterate    ②Elementary school     ③Middle school

④High school     ⑤College or University

7. **Marital status**：   **□**

①Unmarried ②Married ③Divorced

8. **Occupation**：  **□**

①Migrant workers ②Agency/ Institutional personnel

③Business people  ④Students

⑤Unemployed   ⑥Retirees

⑦Other

1. Total household income/year：_________¥

Family population：

10.What do you think of your physical condition： **□**

①Very good  ②Good  ③Average

④Bad ⑤Very bad

11. Whether you have visited a medical institution, taken medication, physiotherapy, or taken leave due to illness or injury in the past two weeks work, school break or bed rest? **□**

①Yes ②No

**IDSHL Scale**

1. If you have hepatitis B (hepatitis B), you should stick to taking antibiotics. **□**

①Agree  ②Disagree ③Don't know

2. When eating shabu lamb, the color of the meat changes from dark to light, which means that it is edible. **□**

①Agree  ②Disagree ③Don't know

3. Hepatitis B can be transmitted sexually. **□**

①Agree  ②Disagree ③Don't know

4. After 2 weeks of treatment for tuberculosis patients, the symptoms of cough and sputum disappeared and their body temperature was normal, indicating that they had been cured. **□**

①Agree  ②Disagree ③Don't know

5. Sick and dead poultry are completely safe to eat after being grilled, steamed, cooked, and cooked. **□**

①Agree  ②Disagree ③Don't know

6. A person who is infected with HIV can tell from the outside. **□**

①Agree  ②Disagree ③Don't know

7. Young adults are in good health, but it is impossible to get tuberculosis. **□**

①Agree  ②Disagree ③Don't know

8. Keep cats, dogs and other pets at home, as long as they do not go out often, they will not bite, and they do not need to vaccinate pets. **□**

①Agree  ②Disagree ③Don't know

9. Free movement is a fundamental human right, and people with infectious diseases should not restrict free movement. **□**

①Agree  ②Disagree ③Don't know

10. If you have the flu (influenza), you should get an injection or take antibiotics as soon as possible. **□**

①Agree  ②Disagree ③Don't know

11. "H1N1" refers to： **□**

①Hepatitis A ②A drug used to treat infectious diseases

③Influenza A ④Don't know

12. Those engaged in catering industry should go to the local health department to apply for: **□**

①Employment certificate ②Health certificate

③Temporary ④Don't know

13.“Infected water”refers to： **□**

①Domestic sewage ②Water body polluted by pathogens

③Water in infectious disease area ④Don't know

14. Children are given the "sugar pill" vaccine to prevent: **□**

①Polio ②Tuberculosis

③Measles ④Don't know

15. “HIV”refers to： **□**

①A bacterium ②A virus

③A parasite ④Don't know

16. Which symptom do you think should be suspected of having hand-foot-and-mouth disease first?

**□**

①Cough and phlegm

②Rash on hands, feet and oral mucosa

③Drink too much and urinate too much

④Don't know

17. People who are susceptible to hand-foot-and-mouth disease： **□**

①Preschool child ②Teenagers

③Young and middle-aged ④Don't know

18. "Vaccine" refers to： **□**

①Biological agents for the treatment of infectious diseases ②Antibiotic

③Biological agents for the prevention of infectious diseases ④Don't know

19. Which of the following ways will spread the hepatitis B virus： **□**

①Shared toothbrush ②Share dishes and utensils

③Shake hands ④Don't know

20. If you get a scratch/bite from a cat/dog, it's very minor, just a little bit of skin. What would you do? **□**

①Rinse the wound with clean water and do not inject rabies vaccine

②Wash the wound immediately with soap and water, and get a rabies vaccine as soon as possible

③The wound was treated with folk earthwork

④Don't know

21. Nail injury requires injection: **□**

①Diphtheria vaccine ②Tetanus vaccine

③Rabies vaccine ④Don't know

22. Where do mosquitoes breed outdoors： **□**

①In ditches, puddles and vessels that hold water ②In the grass or woods

③In the soil of the field ④Don't know

**Cognitive questions**

23. **How long you spend online on average each day**： **□**

①No access ②Less than 1 hour   ③1-2 hours

④2-3 hours ⑤more than 3 hours

24. What are the most important ways you get your health information or knowledge? (Single selection) **□**

①TV ②Internet   ③Radio

④Newspapers and magazines ⑤Family members, colleagues or friends

⑥Medical staff ⑦Others

Material 1

**Drug name:**

**Trade name:** ethylambutol hydrochloride tablets

**Common name:** Tabellae Ethambutoli Hydrochloridi

**Specifications:**

0.25 g

**Indications:**

This product is suitable for the treatment of tuberculosis caused by tuberculosis bacilli in combination with other antituberculosis drugs. It can also be used for the treatment of tuberculous meningitis and atypical mycobacterium infection.

**Usage and dosage:**

1. Adult dosage: combined with other antituberculosis drugs, for the initial treatment of tuberculosis, 15mg/ kg according to body weight, once a day; Or take 25~30mg/ kg orally each time, up to 2.5g, 3 times a week; Or 50mg/ kg up to 2.5g twice a week. Tuberculosis was retreated with 25mg/ kg body weight once a day for 60 days, followed by 15mg/ kg body weight once a day. Atypical mycobacterium infection, 15 to 25mg/ kg daily, once swallowed.

2. Children under 13 years old should not use this product. The dosage for children over 13 years old is the same as that for adults.

**Note:**

1. Interference with diagnosis: Taking this product can increase the serum uric acid concentration.

2. The following conditions should be used with caution: gout, optic neuritis, renal dysfunction.

3. During treatment, eyes, visual field, visual acuity, red and green discrimination should be checked once a day before medication and during the course of treatment, especially for patients with a long course of treatment and a daily dose of more than 15mg/ kg. Serum uric acid determination, because this product can increase the concentration of serum uric acid, cause gout attack, so it should be measured regularly in the course of treatment.

4. If gastrointestinal irritation occurs, ethambutol can be taken with food. The effective blood concentration may not be reached if the daily dose is taken separately, so the daily dose should be taken once.

5. Bacteria can rapidly develop resistance to ethambutol when used alone, so it must be used in combination with other antituberculosis drugs. This product should be used in combination with at least one drug when used in patients who have received antituberculosis drugs.

6. Since there is no practical method to determine blood concentration, the dose should be calculated according to the patient's body weight. In patients with hepatic or renal dysfunction, the blood concentration of this product may increase and the half-life may be prolonged. The dosage should be reduced in patients with renal dysfunction.

**Adverse reaction:**

1. The most common cases are blurred vision, eye pain, red-green color blindness or vision loss, and reduced visual field (it is easy to occur when the daily dose of optic neuritis is more than 25mg/ kg by body weight). Visual changes can be unilateral or bilateral.

2. The less common cases were chills, joint swelling and pain (especially big toe, knee joint), and skin fever and tension on the surface of diseased joints (acute gout, hyperuricemia).

3. The incidence of rashes, fever, arthralgia and other allergic reactions are rare. Or numbness, tingling, burning pain or weakness of the hands and feet (peripheral neuritis).

**Questions：**

25. What tests should be done during treatment with this drug? **□**

a. Visual field, visual acuity, red and green discrimination

① Do ②Don't ③Don't know

b. Serum uric acid measurement **□**

① Do ②Don't ③Don't know

c. Renal function tests **□**

① Do ②Don't ③Don't know

d. Liver function tests **□**

① Do ②Don't ③Don't know

2. What dose should a 15-year-old patient with untreated tuberculosis take if it is taken once a day?**□**

①Take 2 tablets once a day ②Take 3 tablets once a day

③Take 4 tablets once a day ④Take 1 tablet 3 times a day

⑤Don't know

Material 2

Tuberculosis (TB) is a chronic respiratory infection transmitted by droplets emitted when coughing, sneezing or speaking loudly. The main symptoms are coughing, sputum, or blood in sputum for more than 2 weeks. In order to prevent the spread of TB in the school, we will carry out TB prevention and control publicity activities throughout the school. Please cooperate with parents in the following work:

1. If your child or people around you have continuous cough, sputum for more than 2 weeks, or sputum with bloodshot, you should suspect tuberculosis, should immediately go to tuberculosis control professional institutions for examination. There, tuberculosis screening is free and anti-TB drugs are provided if tuberculosis is confirmed.

2. Don't cough or sneeze in close range to others, and open Windows to ventilate frequently to keep indoor air fresh.

3. Educate your children to combine work and rest, maintain adequate nutrition and sleep, more outdoor activities, enhance resistance.

4. If someone in your family or around you has tuberculosis, urge the patient to take medicine on time and adhere to regular treatment. As long as the patient can complete the 6-8 months course of treatment continuously according to the doctor's requirements, more than 90% of the patients can be cured.

**Questions：**

1. Can TB be cured? **□**

①Can ②Cannot

③Don't know

2. What can parents do to prevent the spread of TB in schools?

a. Keep your child well-nourished **□**

①Need to do ②Don't need to do ③Don't know

b. Open Windows frequently for ventilation **□**

①Need to do ②Don't need to do ③Don't know

c. Teach children to exercise more to build up resistance **□**

①Need to do ②Don't need to do ③Don't know

d. Children are forbidden to have outdoor activities **□**

①Need to do ②Don't need to do ③Don't know

**-------------** This is the end of the investigation, thank you for your support!**--------------**

**Investigator's signature** **Supervisor's signature**

**Method of investigation**: ① self-filling ② investigator inquiry

**Survey time:** year month day

**Survey site**:
